# The Conditions Under Which Piracetam Is Used and the Factors That Can Improve National Institute of Health Stroke Scale Score in Ischemic Stroke Patients and the Importance of Previously Unnoticed Factors From a Hospital-Based Observational Study in Taiwan

**DOI:** 10.3390/jcm8010122

**Published:** 2019-01-20

**Authors:** Shu-Yi Chen, Jai-Wen Liu, Yu-Hsun Wang, Jing-Yang Huang, Shiuan-Chih Chen, Shun-Fa Yang, Po-Hui Wang

**Affiliations:** 1 Institute of Medicine, Chung Shan Medical University, 110, Section 1, Chien-Kuo North Road, Taichung 40201, Taiwan; shuyichen2008@gmail.com (S.-Y.C.); sccy399@yahoo.com.tw (S.-C.C.); ysf@csmu.edu.tw (S.-F.Y.); 2 Department of Neurology, Tung’s Taichung MetroHarbor Hospital, No.699, Sec. 8, Taiwan Blvd., Taichung 40201, Taiwan; 3 Department of Emergency Medicine, Chung Shan Medical University Hospital, 110, Section 1, Chien-Kuo North Road, Taichung 40201, Taiwan; jwliuh@gmail.com; 4 Department of Medical Research, Chung Shan Medical University Hospital, 110, Section 1, Chien-Kuo North Road, Taichung 40201, Taiwan; cshe731@csh.org.tw (Y.-H.W.); wchinyang@gmail.com (J.-Y.H.); 5 School of Medicine, Chung Shan Medical University, 110, Section 1, Chien-Kuo North Road, Taichung 40201, Taiwan; 6 Department of Family and Community Medicine, Chung Shan Medical University Hospital, 110, Section 1, Chien-Kuo North Road, Taichung 40201, Taiwan; 7 Department of Obstetrics and Gynecology, Chung Shan Medical University Hospital, 110, Section 1, Chien-Kuo North Road, Taichung 40201, Taiwan

**Keywords:** piracetam, clinical characteristics, acute ischemic stroke, National Institute of Health Stroke Scale, atrial fibrillation, subgroup analysis

## Abstract

This study aimed to explore the associations of piracetam use and the clinical characteristics of NIHSS (National Institute of Health Stroke Scale) changes—the importance of which, as prognosis related factors, was previously unnoticed—and analyze the associations of piracetem with NIHSS changes by stratifying clinical characteristics. This observational retrospective study was conducted by enrolling patients based on 2483 stroke registration data cohorts from a 1200-bed regional Tungs’ Taichung MetroHarbor Hospital, located in central Taiwan from 1 January 1 2011 to 31 December 2015. Patients were excluded if they had intravenous a thrombolytic agent within 3 hours of symptoms onset (*n* = 49), incomplete or erroneous NIHSS scores (*n* = 953), or transient ischemia stroke (*n* = 130). Logistic regression model was applied for associating piracetam treatment and clinical characteristics with NIHSS score changes between admission and discharge, and subgroup analysis to assess the conditions under which piracetam can be used. Multivariate analysis revealed NIHSS scores improvement in atrial fibrillation, large-artery atherosclerosis, underweight, current smoker, ex-smoker, and piracetam. Subgroup analysis showed piracetam is beneficial in the following: age ≥75 years olds, males, those of normal weight, those who are obese, ex-smokers, those with hypertension, dyslipidemia, those without diabetes mellitus, nor atrial fibrillation. The selection of the conditions under which piracetam treatment should be given, and clinical characteristics, is important for NIHSS improvement of ischemic stroke patients in Taiwan.

## 1. Introduction

Ischemic stroke is a major cause of death in developed countries, and the leading cause of long-term disability in survivors. The Ministry of Health and Welfare of Taiwan reported cerebrovascular accidents as the fourth leading cause of death in 2017. A complex series of biochemical reactions occur which may lead to the death of brain cells. Two major types of therapeutic approaches to acute ischemic stroke have been raised [1]. Firstly, thrombolytics were suggested because they could act to restore cerebral blood flow. Current effective treatments for acute ischemic stroke are intravenous thrombolytic therapy within 4.5 h of onset and intra-arterial thrombectomy within 6 h of onset [2]. Secondly, neuroprotectants were suggested because they target cellular biochemical pathways to preserve brain function, enhance neuronal repair, and promote recovery. However, there is no conclusive evidence supporting the use of various other drugs claiming neuroprotective effects [2].

Piracetam (2-Oxo-1-pyrrolidineacetamide), the first representative of the nootropic drugs, is a cyclic derivative of gamma-aminobutyric acid (GABA) [3]. It may increase compromised regional cerebral blood flow and oxygen utilization in brain, and permeability of cell and mitochondrial membrane to intermediates of the Krebs cycle [4]. Piracetam has been found to exert neuroprotective action and offer protection from brain neuron death or cell damage on experimental animals [5,6]. It presents antioxidative antiapoptotic activity and may restore altered neuronal morphology and decrease neuronal density, which were caused by lipopolysaccharides [5]. In addition, piracetam seems to display neuroprotective properties in hemorrhagic shock models [7]. It is approved by the Taiwan Food and Drug Administration for use in acute cardiovascular disease, but the efficacy of piracetam use in patients with acute stroke has yet to be proven. Therefore, in the current environment, and with a better treatment standard, there is room for discussion of the association of piracetam treatment with ischemic stroke patients.

A prespecified hypothesis was therefore raised that piracetam treatment and some clinical variables are associated with ischemic stroke patients’ neurological improvement, as assessed by National Institutes of Health Stroke Scale (NIHSS) score in Taiwan. Their associations with NIHSS improvement, however, are not investigated in Taiwan. Although they were unnoticed previously in Taiwan, the piracetam treatment and the factors that may improve NIHSS score in acute ischemic stroke patients were very important for patient prognosis and should be delineated. The aims of this study were to explore the associations of piracetam use as well as clinical characteristics with NIHSS score changes for acute ischemic stroke patients in Taiwan. In addition, the unique features of this study were to further analyze the conditions under which piracetam can be given to improve NIHSS score by stratifying the epidemiological and comorbidity characteristics.

## 2. Materials and Methods

### 2.1. Source of Data and Study Cohort

**Figure 1:**
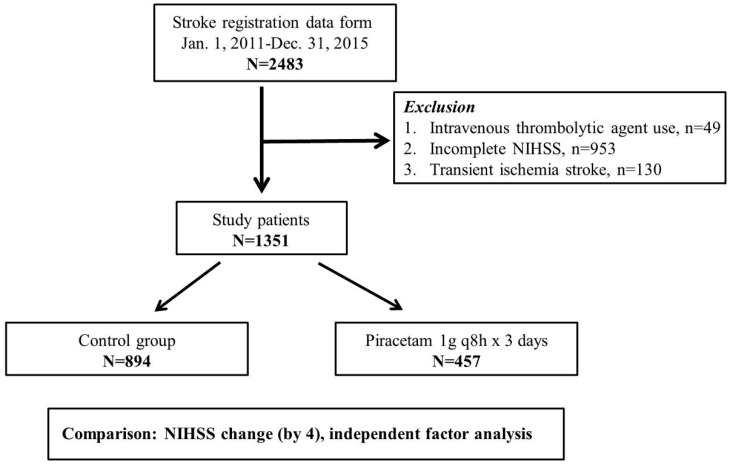
Flowchart of enrollment procedures for the study. A total of 2483 cases were registered during the study period and 1351 patients were recruited into piracetam treatment group and control group after the exclusion of those receiving intravenous thrombolytic agent within 3 h of symptoms onset (*n* = 49), those having incomplete or erroneous admission or discharge NIHSS score (*n* = 953) and those having transient ischemia stroke (*n* = 130). NIHSS, National Institute of Health Stroke Scale.

This observational and retrospective cohort study was conducted by collecting 2483 ischemic stroke patients who were admitted to the intensive care unit (ICU) of Tungs’ Taichung MetroHarbor Hospital, a 1200-bed regional teaching hospital located in central Taiwan, from 1 January 2011 to 31 December 2015, and retrospectively reviewed and analyzed from the stroke registration database of the hospital. The study was conducted in accordance with the Declaration of Helsinki, and the protocol was approved by the Institutional Review Board of Tungs’ Taichung MetroHarbor Hospital (107019, V2/2018-05-17). All subjects gave their informed consent. Criteria for ICU admission were based on recommendations of the American College of Critical Care Medicine and the Society of Critical Care Medicine [8]. Patients were excluded if they had an intravenous thrombolytic agent (recombinant tissue plasminogen activator, rt-PA, Actilyse) within 3 hours of symptom onset (*n* = 49), incomplete or erroneous admission or discharge NIHSS scores (*n* = 953), or transient ischemia stroke (*n* = 130) (Figure 1). Of the 1351 cases included in the study, in addition to basic fluid therapy for these patients, 457 received intravenous piracetam (trade name: Nootropil 200 mg/mL, 5 mL/amp; Nang Kuang Pharmaceutical, Taipei, Taiwan) 1 g every 8 h for 3 days and the remaining 894 did not. The piracetan was not randomized to give. No placebo was given. However, patients were not aware whether piracetam was given or not. The epidemiological and comorbidity characteristics of patients with piracetam and without piracetam were assessed if they were balanced based on standardized difference. This study evaluates the association of piracetam treatment with the NIHSS score changes between the admission and discharge date for patients with acute ischemic stroke.

The NIHSS score was defined previously in [9]. Sample size was calculated based on the analysis for the association of piracetam treatment with NIHSS score change. The alpha error (α) was determined to be 0.05; the beta error (β), 0.2; power, 0.8 (1-beta error). The needed total sample size was 1248, as calculated according to the following formula: H_0_ (null hypothesis): p_1_ = p_2_; H_A_ (alternative hypothesis): p_1_ ≠ p_2_

$$N=\frac{{\left\{Z_{\alpha /2}\sqrt{2\bar{P}\left(1-\bar{P}\right)}+Z_{\beta }\sqrt{p_{1}\left(1-p_{1}\right)+p_{2}\left(1-p_{2}\right)}\right\}}^{2}}{{\left(p_{1}-p_{2}\right)}^{2}}$$

α = 0.05, *Z*_*α*/2_ = 1.96; β = 0.2, *Z_β_* = 0.842
*p*_1_: NIHSS improved rate of piracetam treatment; *p*_2_: NIHSS improved rate of no piracetam treatment; $\bar{P}$ = (*p*_1_ + *p*_2_)/2

### 2.2. Definition of Epidemiological and Comorbidity Variables

The epidemiological and clinical variables include: admission and discharge NIHSS scores, gender, age, hypertension, diabetes mellitus, dyslipidemia, body mass index (BMI), smoking habits, atrial fibrillation, and TOAST (Trial of Organization 10,172 in Acute Stroke Treatment) classification of subtype of acute ischemic stroke [10]. The main evaluators of NIHSS score are Neurology specialists, qualified through training and videotape test based on the National Institute of Neurological Disorders and Stroke (NINDS) trials [11]. BMI values are analyzed according to the World Health Organization (WHO) definition: <18.5 (underweight), 18.5 to 24.9 (normal weight), 25 to 29.9 (overweight), and ≥30 kg/m2 (obese) [12]. Hypertension is defined as systolic blood pressure more than 140 mmHg [13], and diabetes mellitus is diagnosed by fasting blood glucose >126 mg/dL or HbA1c >6.5% (American Diabetes Association, 2010). A system for classification of ischemic stroke was developed for the Trial of Organization 10,172 in Acute Stroke Treatment. The TOAST classification denotes five subtypes of ischemic stroke based on etiology: large-artery atherosclerosis, small-vessel occlusion, cardioembolism, stroke of other determined etiology, and stroke of undetermined etiology [11]. The last two classifications were grouped together in the study. The criteria for dyslipidemia included those who are receiving lipid-lowering drugs or require intervention: low-density lipoprotein >100 mg/dL, or triglyceride >160 mg/dL [14].

For TOAST classification, all patients received an electrocardiogram test, echocardiography, carotid sonography, and transcranial doppler for intracranial large blood vessels flow evaluation. All patients received neuroimaging examination: 90.3% received brain computed tomography (CT), 80% brain magnetic resonance imaging (MRI). Twenty four-hour Holter monitor or electrocardiogram telemetry record for 72 h was done for those suspected of embolic stroke. Based on the above information, a neurologist determines the patient’s TOAST classification. CT perfusion requires hardware and software components that are not widely available. The institution where this study was performed does not have the equipment necessary for CT perfusion. In the determination of TOAST classification, which mainly requires brain MRI and complete cardiovascular evaluation, CT perfusion was not applied. In addition, CT perfusion can evaluate the areas of decreased blood flow, but it is not necessarily compatible with the area of acute infarction being treated at the time. In this study, the patients who did not receive MRI evaluation, of whom acute infarction area could not be determined on a brain CT, or of whom transcranial doppler sonography could not show cerebral artery stenosis, were placed in the fourth classification: undetermined or other causes.

### 2.3. Outcomes

The primary endpoints were to evaluate the associations of piracetam use as well as epidemiological and comorbidity factors with NIHSS score changes and further to determine the conditions under which piracetam improved NIHSS scores. Subtraction of discharge NIHSS score by admission NIHSS score was done. The NIHSS score is a tool used by healthcare providers to objectively quantify the neurological impairment caused by a stroke [15]. A comparison of admission and discharge NIHSS scores greater than 4 at discharge is considered significant improvement [15,16]; otherwise, not improvement.

### 2.4. Statistical Analysis

A standardized difference of more than 10% was used to detect significant piracetam subgroups imbalance between baseline epidemiological and comorbidity variables [17]. Chi-square test was applied to evaluate the associations of piracetam use as well as epidemiological and clinical variables with NIHSS score changes. The multivariate analysis and subgroup analysis were done using logistic regression model with further adjustment for multiple baseline covariates as the piracetam/no piracetam baseline balance might be violated within subgroups. Odds ratios (ORs) and their 95% confidence intervals (CIs) were calculated using logistic regression model. Sensitivity analysis was done for possible interaction among piracetam and other covariates. The adjusted risk (ratio) difference in favor of improvement in NIHSS between piracetam and no piracetam subgroups, and among other variable subgroups, was determined based on logistic regression model. The difference was calculated as I_0_ × (AOR-1), for which AOR is the adjusted odds ratio in the case subgroup and I_0_ the unadjusted odds (instead of incidence in reference 17) for patients in the control subgroup [18]. Therefore, the adjusted ratio difference was calculated as control subgroup odds × (case subgroup AOR-1). All analyses were performed with PASW Statistics 18. All *p* values were 2-sided and a *p* value <0.05 indicated a statistical significance.

## 3. Results

### 3.1. Balance of Piracetam and no Piracetam Subgroups between Baseline Epidemiological and Comorbidity Characteristics

Of the 1351 ischemic stroke patients included in the study, 457 (33.8%) received intravenous piracetam. The mean admission NIHSS score of our database was 6.5 and the NIHSS score was improved in 131 (9.7%) of all patients. Based on the TOAST classification, the patients were grouped into small-vessel occlusion 432 (32.0%), large-artery atherosclerosis 406 (30.0%), embolic stroke 193 (14.3%), and stroke of other etiology 320 (23.7%). The patients who received piracetam treatment were older (piracetam/ no piracetam: 72.0 ± 12.2/70.5 ± 12.5, *p* = 0.031). No statistically significant differences were noted among other categories (Table 1). No significant imbalance was noted between piracetam and no piracetam subgroups for baseline epidemiological and comorbidity variables based on most standardized differences being no more than 10% (Table 1).

### 3.2. The Factors Associated with Ischemic Stroke Based on Univariate Analysis

Univariate analysis was performed for the associations of piracetam use as well as epidemiological and comorbidity variables with NIHSS score changes in ischemic stroke patients (Table 2).

Comparison was made between those with improvement of NIHSS and those without. Univariate analysis showed increased odds ratio in favor of improvement in NIHSS score for age ≥75 years old (OR: 1.88, 95% CI: 1.31–2.71; *p* < 0.001), underweight with BMI <18.5 (OR: 3.37, 95% CI: 1.74–6.52; *p* < 0.001), ex-smoker (OR: 1.73, 95% CI: 1.03–2.91; *p* = 0.039), piracetam treatment (OR: 1.69, 95% CI: 1.18–2.44; *p* = 0.005), atrial fibrillation (OR: 3.14, 95% CI: 2.14–4.62; *p* < 0.001), large-artery atherosclerosis (OR: 2.81, 95% CI: 1.66–4.77; *p* < 0.001), and cardioembolism (OR: 4.19, 95% CI: 2.36–7.43; *p* < 0.001). Hypertension, diabetes mellitus, and dyslipidemia showed no association with NIHSS improvement.

### 3.3. The Factors Associated with Ischemic Stroke Based on Multivariate Analysis

We performed a multivariate analysis of piracetam administration and clinical characteristics on NIHSS improvement in ischemic stroke patients (Table 2). Multivariate analysis showed significant associations of NIHSS score changes with the following subgroups: underweight (OR: 3.05, 95% CI: 1.47–6.30; *p* = 0.003), current smoker (OR: 1.81, 95% CI: 1.07–3.06; *p* = 0.026), ex-smoker (OR: 2.29, 95% CI: 1.22–4.28; *p* = 0.009), piracetam use (OR: 1.73, 95% CI: 1.18–2.55; *p* = 0.005), atrial fibrillation (OR: 3.92, 95% CI: 2.31–8.24; *p* < 0.001), and large-artery atherosclerosis (OR: 2.68, 95% CI: 1.57–4.60; *p* < 0.001).

### 3.4. Sensitivity Analysis for Possible Interaction Test Among Piracetam and Other Covariates, and the Adjusted Ratio Difference in Favor of NIHSS Improvement for These Variables

The sensitivity analysis showed that there was no interaction between piracetam and BMI (*p* = 0.217). *p* values for interactions between piracetam and other variables indicate no interaction if *p* > 0.05 using logistic regression model. No interactions were found among piracetam and variables other than BMI such as age (*p* = 0.691), gender (*p* = 0.779), smoking habits (*p* = 0.713), hypertension (*p* = 0.292), diabetes mellitus (*p* = 0.622), dyslipidemia (*p* = 0.765), atrial fibrillation (*p* = 0.605), and TOAST (*p* = 0.942). Therefore, any variables were unnecessary to exclude from multivariate analysis in Table 2 to re-associating piracetam and clinical variables with NIHSS score changes for sensitivity analysis.

Regarding the significant associations of piracetam and clinical variables with NIHSS score improvement by multivariate analysis in Table 2, adjusted ratio difference was calculated in the item with statistically significant comparison with its reference of each clinical category (Table 3). Of the 1351 ischemic stroke patients included in this study, NIHSS scores were improved in 131 cases (9.7%) and the odds were 0.11 (131/1220). If no piracetam was given, only 8.1% (72/822 + 72) stroke patients had improved NIHSS scores and the odds were 0.09 (72/822). Piracetam improved the NIHSS scores in 12.9% (59/398 + 59) of patients and the odds increased to 0.15 (59/398; an increase of odds 0.06 (0.15–0.09)). In the analysis of the adjusted ratio difference in favor of improvement in NIHSS, piracetam treatment had 6.4% (95% CI: 2.2%–13.6%) increase for improvement of ischemic stroke as compared to no piracetam (Table 3). Other conditions that had this advantage, compared to their control conditions, were underweight 20.3% (95% CI: 4.7%–52.2%), current smoker 7.5% (95% CI: 0.6%–18.9%), ex-smoker 11.9% (95% CI: 2.0%–30.2%), and large-artery atherosclerosis 8.6% (95% CI: 2.9%–18.4%). However, the factor most associated with NIHSS improvement for stroke patients was atrial fibrillation 23.4% (95% CI: 2.5%–57.9%) (Table 3).

### 3.5. Subgroup Analysis

**Figure 2:**
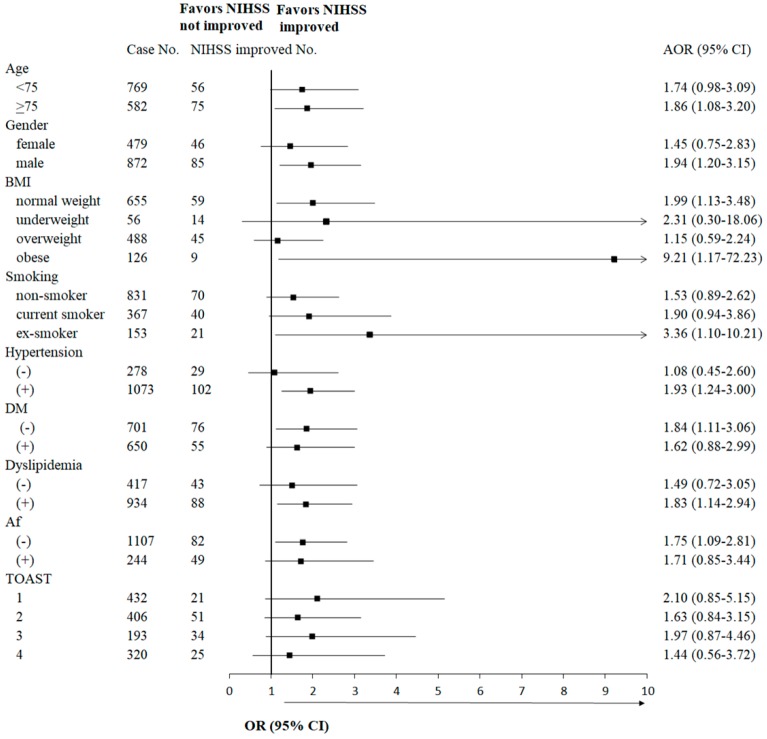
Subgroup analysis of epidemiological and comorbidity variables for the associations of piracetam with NIHSS score improvement. Association of piracetam treatment with NIHSS improvement was evaluated in each subgroup in each category. Logistic regression model was used for statistical analysis. NIHSS, National Institute of Health Stroke Scale; BMI, body mass index; DM, diabetes mellitus; Af, atrial fibrillation; TOAST, Trial of Organization 10,172 in Acute Stroke Treatment classification; 1, small-vessel occlusion; 2, large-artery atherosclerosis; 3, cardioembolism; 4, others; (−), negative; (+), positive; AOR, adjusted odds ratio, adjusted among the categories including age, gender, BMI, smoking habit, hypertension, DM, dyslipidemia, Af and TOAST; 95% CI, 95% confident interval.

Using subgroup analysis of clinical variables, piracetam treatment improving NIHSS scores was found when it was added in the following conditions (Figure 2 and Table 3): age ≥75 years old (OR: 1.86, 95% CI: 1.08–3.20, *p* = 0.025), male (OR: 1.94, 95% CI: 1.20–3.15, *p* = 0.007), normal weight (OR: 1.99, 95% CI: 1.13–3.48, *p* = 0.016), obesity (OR: 9.21, 95% CI: 1.17–72.23, *p* = 0.035), ex-smoker (OR: 3.36, 95% CI: 1.10–10.21, *p* = 0.033), hypertension (OR: 1.93, 95% CI: 1.24–3.00, *p* = 0.004), no diabetes mellitus (OR: 1.84, 95% CI: 1.11–3.06, *p* = 0.018), dyslipidemia (OR: 1.83, 95% CI: 1.14–2.94, *p* = 0.013), and no atrial fibrillation (OR: 1.75, 95% CI: 1.09–2.81, *p* = 0.020). The different TOAST classifications showed no obvious favorable OR in NIHSS improvement, while adding piracetam (Figure 2 and Table 3).

## 4. Discussion

Piracetam treatment could be associated with NIHSS improvement in ischemic stroke patients in Taiwan based on univariate and multivariate analyses in this study. The epidemiological and comorbidity characteristics of patients with piracetam and without piracetam were balanced based on standardized difference. Age is an important variable and has been used for the evaluation of the effects of intravenous thrombolysis with alteplase for acute ischemic stroke [19]. Although the standardized difference of age in this study was 0.130 (13%) and beyond 10%, it was below 0.2 (20%), which indicates a 15% of non-overlap in the two age subgroups distribution. It has been reported that an effect size is associated with three different measures of non-overlap between two populations and effect size of 0.2 represents a small effect size [20,21,22].

A study by Piracetam in Acute Stroke Study (PASS) in 1997 showed improvement in neurological symptoms and Barthel Index scores at 12 weeks, but there was no effect in 1-month mortality [23]. Cochrane Review pointed out that piracetam is ineffective in patients with presumed ischemic stroke, although other potential beneficial effects of piracetam remain unclear because of insufficient well-controlled studies [24]. Experiments in animals suggested that piracetam could have beneficial effects in patients with acute stroke [25]. A meta-analysis of studies in models of stroke/cerebral ischemia in rats also supported the potential effectiveness of piracetam [26]. Piracetam has been demonstrated to increase brain penetration in ischemic stroke rats and significantly decrease brain infarct volume [27]. However, it has been approved for use by the Taiwan National Health Insurance Administration. Piracetam was reported to increase cerebral blood flow and glucose metabolism in both infarcted and penumbral tissues and to be an effective adjunct of verbal skills in stroke patients with aphasia (confirmed by neuroimaging tests) [28,29,30], but it failed to improve visuospatial and recognition memory, and cognitive functions such as reasoning [31,32]. Other than standard supportive treatments, neurologists often wish there were ways to improve outcomes in acute ischemic stroke patients who are presented to the hospital more than 4.5 hours after onset of symptoms, do not qualify for intravenous thrombolysis, intraarterial thrombectomy, or suffer from deterioration of clinical presentation.

To date, only risk factors, but no clinical characteristics that are associated with NIHSS score changes, are reported in ischemic stroke in Taiwan. Age, hypertension, atrial fibrillation, smoking habit, glucose intolerance, and hypercholesterolemia have been reported to be risk factors for ischemic stroke according to the Hisayama study [33]. The unique features of this study include the investigation of baseline epidemiological and comorbidity characteristics as variables that may affect NIHSS score changes, but not as risk factors of ischemic stroke. Once acute ischemic stroke occurred, underweight, current smoker, ex-smoker, atrial fibrillation, and large-artery atherosclerosis were associated with NIHSS improvement in addition to piracetam use, based on the multivariate analysis in this study.

Another unique feature of this study demonstrated that piracetam is beneficial in the following: age ≥75 years old, male, normal weight or obese, ex-smoker, hypertension, dyslipidemia, no diabetes mellitus, and no atrial fibrillation by subgroup analysis. It implies that although ischemic stroke patients with atrial fibrillation have better NIHSS improvement, as compared to those without atrial fibrillation, patients without atrial fibrillation may have improved NIHSS score after piracetam treatment. The ischemic stroke patients may have similar association after they take piracetam treatment in the following conditions: normal weight and obesity. Piracetam may enhance this association in ex-smoker patients. This shows that ischemic stroke patients, who have multiple risk factors and are under the current treatment in a specialized ward, do not decrease the chances of substantial improvement [34]. Piracetam can improve ratio difference for NIHSS by 6.4%. However, atrial fibrillation can most obviously improve this difference up to 23.4% among the above variables.

## 5. Limitations

The limitations of this study include there is no way to standardize the use or dosage of oral piracetam after 3 days intravenous form injection and no follow-up analysis for a longer time period. There was no follow-up brain imaging in all patients, so it is impossible to determine whether piracetam injection increases the incidence of cerebral hemorrhage or other sequales that would affect neurological evaluation [35]. For patients with more severe ischemic stroke (NIHSS score >10) [36], piracetam might increase the detection of NIHSS improvement but could not guarantee decrease of overall dependence or disability. The dosage used in this study is based on the manufacturer’s suggestion and it is appreciably different from the dose used in the PASS [23]. After classification of each clinical variable, the sample size of each subgroup may not be large enough to draw conclusions.

## 6. Conclusions

Piracetam, underweight, current smoker, ex-smoker, atrial fibrillation, and large-artery atherosclerosis increase the odds ratio of NIHSS improvement for ischemic stroke patients in Taiwan. Even if patients do not meet above conditions, subgroup analysis reveals that piracetam may improve NIHSS score for ischemic stroke patients under the following conditions: age ≥75 years old, male, normal weight, obesity, ex-smoker, hypertension, no diabetes mellitus, dyslipidemia, or no atrial fibrillation. Additionally, piracetam may enhance the NIHSS improvement in ex-smoker patients. Thus, the selection of the conditions under which piracetam should be given, and clinical characteristics, is very important for NIHSS improvement of ischemic stroke patients in Taiwan. This hospital-based observational study reveals that these factors and conditions are important for the NIHSS score changes for the ischemic stroke patients in Taiwan, which was unnoticed previously. With the above limitations, it is necessary to increase the sample size to verify whether the use of piracetam during the acute stage of ischemic stroke really decreases NIHSS score. Especially after each clinical variable is subdivided, the smaller numbers of cases may not provide sufficient power for evaluating the OR in this specific group of patients. Furthermore, the mechanisms, which are involved in the association of piracetam with improved NIHSS, should be investigated and delineated in the future.

## Figures and Tables

**Table 1:** Baseline epidemiological and comorbidity characteristics of ischemic stroke patients receiving piracetam versus those not receiving piracetam in Taiwan.

Characteristics	Piracetam *n* = 457 (%)	No Piracetam *n* = 894 (%)	*p* Value	Standardized Difference
Age (years)				
mean ± SD	72.0 ± 12.2	70.5 ± 12.5	0.031	0.130
Age (years)				
<75	246 (53.8%)	523 (58.5%)	0.101	0.095
≥75	211 (46.2%)	371 (41.5%)		
Gender				
male	295 (64.6%)	577 (64.5%)	0.960	0.002
female	162 (35.4%)	317 (35.5%)		
BMI				
underweight	17 (3.8%)	39 (4.5%)	0.564	0.035
normal weight	243 (52.0%)	421 (48.1%)		0.078
overweight	160 (35.6%f)	328 (37.5%)		0.039
obese	39 (8.7%)	87 (9.9%)		0.041
Smoking				
non-smoker	272 (59.5%)	559 (62.5%)	0.179	0.061
current smoker	138 (30.2%)	229 (25.6%)		0.103
ex-smoker	47 (10.3%)	106 (11.9%)		0.051
Hypertension				
negative	88 (19.3%)	190 (21.3%)	0.390	0.050
positive	369 (80.7%)	704 (78.7%)		
Diabetes mellitus				
negative	252 (55.1%)	449 (50.2%)	0.087	0.094
positive	205 (44.9%)	445 (49.8%)		
Dyslipidemia				
negative	127 (27.8%)	290 (32.4%)	0.080	0.100
positive	330 (72.2%)	604 (67.6%)		
Atrial fibrillation				
negative	370 (81.0%)	737 (82.4%)	0.505	0.036
positive	87 (19.0%)	157 (17.6%)		
TOAST				
small-vessel occlusion	135 (29.5%)	297 (33.2%)	0.558	0.080
large-artery atherosclerosis	140 (30.6%)	266 (29.8%)		0.017
cardioembolism	70 (15.3%)	123 (13.8%)		0.043
others	112 (24.5%)	208 (23.3%)		0.028

Statistical analysis: chi-square test and Student’s *t* test. Abbreviations: SD, standard deviation; BMI, body mass index; TOAST, Trial of Organization 10,172 in Acute Stroke Treatment classification.

**Table 2:** Univariate and multivariate analyses for the associations of piracetam treatment as well as epidemiological and comorbidity factors with changes of NIHSS score in ischemic stroke patients in Taiwan.

Variables	Group A–NIHSS Improved (>4) *n* = 131 (%)	Group B–NIHSS Not Improved (≤4) *n* = 1220 (%)	Univariate Analysis OR (95% CI)	*p* Value	Multivariate Analysis OR (95% CI)	*p* Value
Age (years)						
<75	56 (42.7%)	713 (58.4%)	1.00	<0.001	1.00	0.053
≥75	75 (57.3%)	507 (41.6%)	1.88 (1.31–2.71)		1.52 (1.00–2.33)	
Gender						
male	46 (35.1%)	443 (35.5%)	1.00	0.932	1.00	0.437
female	85 (64.9%)	787 (64.5%)	1.02 (0.70–1.48)		0.82 (0.50–1.35)	
BMI						
underweight	59 (46.5%)	596 (49.7%)	1.00	0.002	1.00	0.026
normal weight	14 (11.0%)	42 (3.6%)	3.37 (1.74–6.52)	<0.001	3.05 (1.47–6.30)	0.003
overweight	45 (35.4%)	443 (37.0%)	1.03 (0.68–1.54)	0.901	1.11 (0.72–1.70)	0.644
obese	9 (7.1%)	117 (9.8%)	0.78 (0.38–1.61)	0.498	0.93 (0.44–2.00)	0.858
Smoking						
non-smoker	70 (53.4%)	761 (62.4%)	1.00	0.083	1.00	0.017
current smoker	40 (30.5%)	327 (26.8%)	1.33 (0.88–2.00)	0.172	1.81 (1.07–3.06)	0.026
ex-smoker	21 (16.0%)	132 (10.8%)	1.73 (1.03–2.91)	0.039	2.29 (1.22–4.28)	0.009
Piracetam						
negative	72 (55.0%)	822 (67.4%)	1.00	0.005	1.00	0.005
positive	59 (45.0%)	398 (32.6%)	1.69 (1.18–2.44)		1.73 (1.18–2.55)	
Hypertension						
negative	29 (22.1%)	249 (20.4%)	1.00	0.642	1.00	0.465
positive	102 (77.9%)	971 (79.6%)	0.90 (0.58–1.39)		0.84 (0.52–1.35)	
Diabetes mellitus						
negative	76 (58.0%)	625 (51.2%)	1.00	0.141	1.00	0.468
positive	55 (42.0%)	595 (48.8%)	0.76 (0.53–1.10)		0.86 (0.58–1.29)	
Dyslipidemia						
negative	43 (32.8%)	374 (30.7%)	1.00	0.610	1.00	0634
positive	88 (67.2%)	846 (69.3%)	0.91 (0.62–1.33)		1.11 (0.37–1.69)	
Atrial fibrillation						
negative	82 (62.6%)	1025 (84.0%)	1.00	<0.001	1.00	<0.001
positive	49 (37.4%)	195 (16.0%)	3.14 (2.14–4.62)		3.92 (2.31–8.24)	
TOAST						
small-vessel occlusion	21 (16.0%)	411 (33.7%)	1.00	<0.001	1.00	<0.001
large-artery atherosclerosis	51 (38.9%)	355 (29.1%)	2.81 (1.66–4.77)	<0.001	2.68 (1.57–4.60)	<0.001
cardioembolism	34 (26.0%)	159 (13.0%)	4.19 (2.36–7.43)	<0.001	0.87 (0.38–1.96)	0.933
others	25 (19.1%)	295 (24.2%)	1.66 (0.91–3.02)	0.098	1.06 (0.56–2.02)	0.640

Statistical analysis: chi-square test and logistic regression model. Abbreviations: NIHSS, National Institute of Health Stroke Scale; BMI, body mass index; TOAST, Trial of Organization 10,172 in Acute Stroke Treatment classification; OR, odds ratio; 95% CI, 95% confidence interval.

**Table 3:** Subgroup analysis of clinical variables for the associations of piracetam with NIHSS score improvement and the adjusted ratio difference in each clinical category in Taiwan ischemic stroke patients.

Variables	NIHSS Improved (*n* = 131)	NIHSS NotImproved (*n* = 1220)	Rate (Proportion)	95% CI Lower-Upper Rate	SE	*p* Value *	Adjusted Ratio Difference of NIHSS Improvement (95% CI) ^†^
Age (years)							
<75	56	713	0.073	0.054–0.091	0.009	0.061	-
≥75	75	507	0.129	0.102–0.156	0.014	0.025	
Gender							
male	46	433	0.096	0.070–0.122	0.013	0.273	-
female	85	787	0.097	0.078–0.117	0.010	0.007	
BMI							
normal weight	59	596	0.090	0.068–0.112	0.011	0.016	reference
underweight	14	42	0.250	0.137–0.363	0.058	0.425	20.3% (4.7%–52.2%)
overweight	45	443	0.092	0.067–0.118	0.013	0.688	-
obese	9	117	0.071	0.026–0.116	0.023	0.035	-
Smoking							
non-smoker	70	761	0.084	0.065–0.103	0.010	0.123	reference
current smoker	40	327	0.109	0.077–0.141	0.016	0.074	7.5% (0.6%–18.9%)
ex-smoker	21	132	0.137	0.083–0.192	0.028	0.033	11.9% (2.0%–30.2%)
Piracetam							
negative	72	822	0.081	0.063–0.098	0.009	-	reference
positive	59	398	0.129	0.098–0.160	0.016		6.4% (2.2%–13.6%)
Hypertension							
negative	29	249	0.104	0.070–0.140	0.018	0.866	-
positive	102	971	0.095	0.078–0.113	0.009	0.004	
Diabetes mellitus							
negative	76	625	0.108	0.085–0.131	0.012	0.018	-
positive	55	595	0.085	0.063–0.106	0.011	0.124	
Dyslipidemia							
negative	43	374	0.103	0.074–0.132	0.015	0.280	-
positive	88	846	0.094	0.075–0.113	0.010	0.013	
Atrial fibrillation							
negative	82	1025	0.074	0.059–0.090	0.008	0.020	reference
positive	49	195	0.201	0.151–0.251	0.026	0.131	23.4% (2.5%–57.9%)
TOAST							
Small-vessel occlusion	21	411	0.049	0.028–0.069	0.010	0.106	reference
large-artery atherosclerosis	51	355	0.126	0.093–0.158	0.016	0.145	8.6% (2.9%–18.4%)
cardioembolism	34	159	0.176	0.122–0.230	0.027	0.106	-
others	25	295	0.078	0.049–0.108	0.015	0.453	-

Statistical analysis: logistic regression model. * *p* value, to define the statistical significance for the associations of piracetam with NIHSS score improvement in each subgroup of each clinical category in subgroup analysis using logistic regression model. ^†^ The adjusted ratio difference in favor of improvement in NIHSS that was calculated as I_0_ × (AOR-1), for which AOR is the adjusted odds ratio in case subgroup and I_0_ the unadjusted odds for patients in control subgroup. The adjusted ratio difference was only calculated in the item with statistically significant comparison with its reference of each clinical category using multivariate analysis in Table 2, otherwise it was not showed. Abbreviations: NIHSS, National Institute of Health Stroke Scale; BMI, body mass index; TOAST, Trial of Organization 10,172 in Acute Stroke Treatment classification; SE, standard error; 95% CI, 95% confidence interval.
